# Mercy Hospital Medical Report—Capillary Congestion of Brain, with Cerebral Hyperæsthesia—Acute Suppression of Urine, with Hiccough

**Published:** 1879-11

**Authors:** H. M. Starkey

**Affiliations:** House Physician


MEDICAL REPORT. Service of DRS. N. S. and F. H. DAVIS.

Reported by H. M. STARKEY, M. D., House Physician.

*Case 1.*—J. S., German, age 40, bachelor, peddler. Entered hospital April 19, 1879.

The only history obtainable from friends is as follows: Patient has always been very healthy, and retired last night apparently in perfect health. Was found in bed at half past six this morning, breathing heavily and frothing from the mouth. Within a few minutes he had a slight convulsive seizure, which stiffened most of the muscles without producing decided spasm. Was taken to the hospital two blocks distant, and first seen by the house doctor about half past seven a.m.

As brought in, his condition was so alarming that it was thought by the attendant that he would not live till he could be placed in bed. When first seen by house physician, patient was lying unconscious upon his back, forehead and nose rather pale, while lips and lower part of face were livid, and lips were covered with foam. There was now apparently neither paralysis nor spasm of any part. Breathing slow, fifteen per minute, labored and stertorous, and lungs seemed almost full of mucus, as coarse râles could be easily heard from all parts of the lungs. Pulse full and slow, and heart beating very laboriously. All vessels quite full, those of the head not particularly so. Pupils a little contracted, yet respond sluggishly to the light.

In a few moments, and during the examination, a severe attack of dyspnœa supervened, lasting about a minute. During this attack patient was in extreme distress, gasping for breath, writhing and struggling to get out of bed. Face became purple, and ears and lips almost black. Finally, when it seemed as if he could no longer live, his breath came freely, and he was at once relieved. During this spasm the pulse rose to 130, but fell to about 100 after the paroxysm passed. Cold was applied to the head and sinapisms to the calves of the legs. He remained comfortable about three minutes, when the paroxysm returned with all the phenomena before described. There was a little vomiting about this time, the vomited matter being thin and yellowish. Also unconscious discharges of urine and fæces. The attacks of dyspnœa continued to occur every three to five minutes for about an hour, gradually decreasing in severity. About half past nine patient passed into a comparatively quiet sleep, which lasted an hour or more, when he suddenly awoke in the wildest delirium. So violent was the delirium that it was found necessary at once to confine his hands, and even then it required two nurses to use their utmost strength to restrain him in bed. He struggled tremendously and yelled and screamed uproariously. Chloroform was administered to the anæsthetic degree, but this quieted him only so long as the anæsthetic influence was exerted, and within two minutes after the administration was discontinued, he was as uncontrolable as before. The anæsthesia was repeated several times, but with the same result.

As nothing could be given by the mouth, chloral hydrate ʒss. and potass. bromide gr. xx was administered every half hour in enema. This producing no appreciable effect, in two hours the same medicines, with the addition of ext. veratriæ fl. gtt. j, was given every half hour by the stomach.

The patient being anæsthetized, and a tube passed back of the glottis and from seven to ten centimeters into the œsophagus, the medicine held in milk or beef broth was injected with a syringe through this tube. This was continued till five o'clock p.m., and a number of administrations were made at irregular intervals after that time, and before nine o'clock, but although all were retained, they seemed not to have the slightest effect. In every instance the patient became wild as before almost immediately after the chloroform was withdrawn, and remained in this condition till he was again anæsthetized. In several instances he was kept under the influence of the anæsthetic for twenty or thirty minutes, thinking this, with the aid of the chloral, might so far quiet as to allow sleep, but no permanent effect was produced. Not only would the patient not sleep himself, but he baffled all attempts at sleep on the part of other patients, and his yells could be heard over a block away.

Other means having failed, at ten p. m. the right temporal artery was opened, and about 640 C. C. of blood withdrawn. Patient became more quiet before the blood ceased flowing, and within half an hour the pulse, which had been from 120 to 130, fell to 88, and patient became quiet and rational enough to nod his head when asked if he was thirsty, and to open his mouth when water was offered. He swallowed without difficulty, and shook his head when asked if his head ached. At this time he kept moving his head rapidly from side to side, but within another half-hour passed into a calm and quiet sleep. He slept most of the time till morning, awaking about every half-hour; he tossed restlessly for a few seconds, and then again went to sleep. His pulse remained between 85 and 95.

April 20. — Slept quietly most of the day, awaking occasionally and talking coherently, yet seeming a little dazed and confused withal.

Was given fluid extract of ergot and tincture of physostignas āā grams 2 every four hours. Pulse remains quiet and about eighty beats per minute.

April 21. — Continues to improve. Talks naturally about himself and his business, and gave directions for the custody of property. Close observation shows a slight degree of paresis of the right side of the body and of the right extremities and of the left side of the face. The tongue, when protruded, points a little to the left of the median line. The right hand has not so powerful a grip as its fellow, although the man is right-handed; and neither the tactile sense nor the sense of tickling is as acute on the right as on the left side.

April 22. — Felt so well that he was allowed to rise and dress and to walk about the ward. He concluded to go out and see about his business, and accordingly went down town and was gone nearly all day. Returned at evening without having suffered inconvenience from his prolonged exertion.

April 23. — Sat up all day and walked about the ward and yard much of the time. Complains of a dull, heavy feeling of fullness about the head.

April 24. — Was out of the hospital most of the day, and seems nearly well in every respect, but says he will not be well till he has been bled *more thoroughly*; that if we would now take away twice as much blood as was before withdrawn, he would be all right. As he bears the marks of numerous previous cuppings and bleedings, and says he has been in the habit of being bled every few months, it is probable that further abstraction of blood might prove serviceable.

April 25. — Still improving, but complains of a sense of fullness and oppression, particularly about the head. Bled from left arm 320 to 380 C.C., after which he felt much better.

April 27. — Left hospital apparently well.

*Remarks.* — This was, doubtless, a case of severe capillary congestion of the brain, with cerebral hyperæsthesia. The prompt and complete relief obtained by *blood-letting* illustrates the great value of that *unfashionable* remedy in a certain class of cases.

*Case II.—Acute Suppression of Urine, with Hiccough.*

E. O. Irish, aged 40, expressman, entered the hospital April 18, 1879.

*History.* — History, which was not obtained till three days later is as follows: Two weeks ago patient was riding upon wagon, perspiring freely and feeling well. Wind changed suddenly, and blew cold from the lake. Felt chilled, and an immediate and uncontrollable desire to urinate, but upon trial it was found impossible to do so. Has since passed very little water, and about a week after the chill was seized with hiccough, which has continued almost incessantly, giving no rest day or night since.

*Present Condition.* — Seems very much exhausted from lack of rest and continuous wearing of the hiccough. Temperature nearly normal, 37.4° C.; pulse 100, and moderately strong. Skin rather dry and harsh. Thirst is intense, and he has some appetite, which it is impossible to gratify, as every attempt at swallowing increases the spasmodic action to such an extent that vomiting is produced.

Administered ten grains each of chloral hydrate and gum camphor, which gave relief in a few minutes, and allowed restful sleep for two hours, when the hiccough returned. By repeating the dose a number of times, a comparatively comfortable night was obtained.

March 19 and 20. Hiccough continues with short intermissions. Chloral and camphor give but temporary relief, under largely increasing doses. Tried, without effect, heat to the epigastrium, the elastic bandage applied about the chest and abdomen, and various drugs. Patient is eating a little while under the effect of the chloral mixture, but a few swallows is sufficient to cause return of the spasmodic action.

March 21. Urine, which is rather scanty, is not found to contain albumen or other abnormal constituent, but the normal ingredients are all much reduced, and the specific gravity is but 1006. Ordered a warm bath at evening. Fl. ext. jaborandi m. 20, every four hours, alternating with a teaspoonful of the following mixture every four hours:

| | GRAMS. |
|---|---|
| ℞ Potass. brom.................................. | |
| " acetatis āā.................................. | 16 |
| Vini colchici rad.................................. | 16 |
| Syrupi et aquæ āā q. s. ad.................. | 128 |

M.

March 22. Very profuse perspiration. Four hours comfortable sleep in early part of night. Much distress since, hiccough being quite continuous. Secretion of urine increasing a little.

March 23. Sweating still copious, and secretion of urine is *very* free, as much as three pints being collected in twelve hours. Specific gravity of urine 1009, and it contains neither albumen nor sugar.

Notwithstanding re-establishment of the secretions, hiccough continues almost incessantly, and patient, taking little nourishment, is becoming more exhausted. This continued, with little modification effected by treatment, until April 5, when patient died of exhaustion.

Autopsy not allowed.

---

CONSTITUTION AND PROPERTIES OF DIALYSED IRON. (M. Personne, *Acad. de Méd.*, Aug. 5-10-19, 1879. *Lyon Médical*, Sept. 1879, p. 19.)

The ferruginous liquor known as dialysed iron is not a veritable aqueous solution of sesquioxide of iron; it is only a pseudo-solution of sesquioxide of iron modified, differing from the ordinary oxide in that it is insoluable in acids, and has less specific heat. It was discovered more than twenty-five years ago by M. Péan de Saint-Gilles, and since Graham has proved that this modified sesquioxide of iron is a colloid body, that is to say not capable of forming a true solution. Its apparent solution does not possess the property of traversing organic membranes. It is insoluable in the gastric juice, and even in the most energetic acids, it cannot be absorbed, and in consequence is completely inactive.

M. Berthelot had nothing to add to what M. Personne had said, except that if he had to choose a preparation which had no effect on the economy, he would take dialysed iron.

